# Broadband Sub-Micron Moth-Eye Anti-Reflection Coatings on Silicon for Wafer-Level CMOS–SOI–MEMS Thermal Infrared Sensors

**DOI:** 10.3390/mi17020170

**Published:** 2026-01-28

**Authors:** Moshe Avraham, Yael Nemirovsky

**Affiliations:** Electrical and Computer Engineering Department, Technion–Israel Institute of Technology, Haifa 320003, Israel; smoa@campus.technion.ac.il

**Keywords:** moth-eye, anti-reflection coating, long-wave infrared (LWIR), wafer-level packaging, CMOS–SOI–MEMS sensors, silicon infrared windows, broadband optical transmission, angle-tolerant optics, FDTD, FTIR

## Abstract

Silicon windows in wafer-level packaged LWIR sensors suffer ~30% Fresnel reflection per interface, limiting optical throughput and detector sensitivity. We present an end-to-end design, fabrication, and validation framework for CMOS-compatible moth-eye anti-reflection coatings patterned directly on silicon wafers. Our approach integrates the effective medium theory, a transfer matrix analysis, full-wave FDTD simulations, and experimental Fourier-transform infrared (FTIR) measurements to optimize subwavelength pillar arrays for broadband (8–14 μm) and angle-tolerant performance. Fabricated structures demonstrate a 46.7% responsivity boost in CMOS–SOI–MEMS thermal sensors compared to bare silicon windows, while simulations predict up to 85.1% transmission and 57.1% responsivity enhancement for double-sided patterning. These results establish moth-eye metasurfaces as a scalable, CMOS-compatible solution for next-generation wafer-level processing and packaging infrared sensing platforms, transforming optical improvements into measurable electrical performance gains. The contribution of this work is the end-to-end framework for designing moth-eye wafer level processing and packaging for “real-life” CMOS-compatible infrared sensors manufacturing.

## 1. Introduction

Long-wave infrared (LWIR) sensors are critical for thermal imaging, environmental monitoring, and defense applications, yet their performance is often limited by optical losses in wafer-level packaging [1,2]. Silicon windows, widely used for hermetic sealing in CMOS–SOI–MEMS sensors, exhibit ~30% Fresnel reflection per interface, reducing optical throughput and detector sensitivity. Conventional multilayer anti-reflection coatings (ARCs), while effective in the visible and near-IR spectra, present significant challenges for LWIR applications due to their narrowband nature and integration constraints [3,4,5]. Specifically, these coatings are difficult to implement in wafer-level MEMS packages and are often incompatible with standard CMOS fabrication flows because of thermal budget limitations and material mismatches.

Subwavelength “moth-eye” nanostructures offer a promising alternative by creating a gradual refractive index transition between air and silicon, enabling broadband impedance matching with wide angular acceptance [4,6,7]. While extensively studied for visible optics, their implementation in the LWIR band—particularly using CMOS-compatible, wafer-scale fabrication—remains largely unexplored. This gap is critical for silicon-based infrared sensors, where optical transmission directly impacts electrical responsivity.

In this work, we present a comprehensive framework for designing and validating moth-eye anti-reflection coatings patterned directly on silicon wafers. Our approach combines effective medium theory, transfer matrix analysis, full-wave FDTD simulations, and experimental Fourier-transform infrared FTIR measurements to optimize subwavelength pillar arrays for the 8–14 μm band. Unlike prior studies focused solely on optical metrics, we quantify system-level impact by correlating transmission gains with responsivity improvements in CMOS–SOI–MEMS thermal sensors. Fabricated structures achieve a 46.7% responsivity boost, while simulations predict up to 85.1% transmission and 57.1% enhancement for double-sided patterning. These results establish moth-eye metasurfaces as a scalable, CMOS-compatible solution for next-generation wafer-level infrared sensing platforms.

This work addresses critical gaps in prior moth-eye research for infrared applications. While previous studies have demonstrated transmission improvements in the visible and near-IR spectra, they typically focus exclusively on optical efficiency metrics without analyzing diffraction behavior, angular scattering, or system-level electrical performance. Furthermore, conventional multilayer thin-film anti-reflection coatings (e.g., quarter-wave stacks of SiO_2_/Si_3_N_4_ or ZnS/YF_3_), though effective over narrow bands, suffer from chromatic dispersion, limited angular tolerance, and present significant integration challenges for MEMS devices due to thermal budget constraints and material compatibility issues [3,4,5,8,9,10]. Moth-eye structures, by contrast, are formed by subtractive patterning of the substrate itself, eliminating material interfaces and offering intrinsically broadband performance governed by geometry rather than interference.

Here, we present a comprehensive framework that integrates effective medium theory, full-wave diffraction simulations, and experimental validation to quantify not only optical transmission but also the direct impact on electrical responsivity in CMOS–SOI–MEMS thermal sensors. This holistic approach—spanning subwavelength modeling through wafer-level integration constraints—establishes moth-eye metasurfaces as a viable manufacturing solution rather than a laboratory demonstration.

## 2. Optical Theory and Modeling Framework

### 2.1. Silicon Wafer-Level Packaging and the Role of Anti-Reflection Coatings

**Figure 1:**
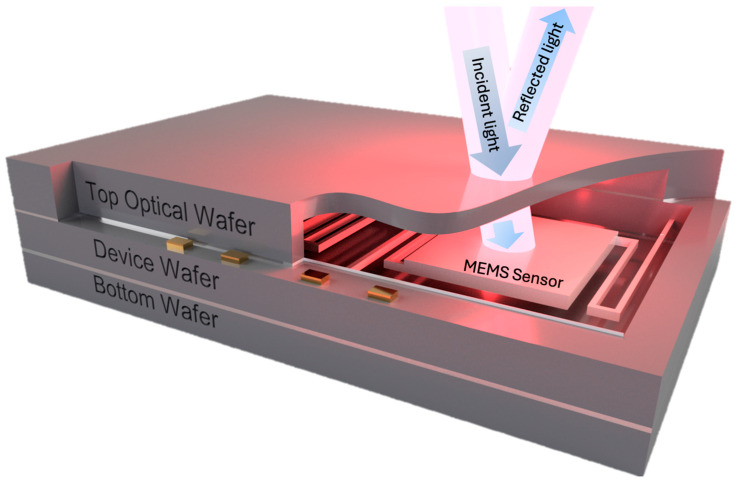
Schematic of the wafer-level package architecture for a high-vacuum thermal IR MEMS sensor. The top optical silicon wafer enables vacuum encapsulation. While silicon is transparent in the LWIR region, it introduces significant optical reflection.

A key component in many infrared sensor architectures, including CMOS–SOI–MEMS devices such as the TMOS detector, is the wafer-level package (WLP) [11]. The package provides hermetic sealing, mechanical protection, and high-vacuum encapsulation, which is essential to reduce thermal conduction and improve responsivity [12,13,14,15,16]. In this architecture, a silicon cap wafer is typically used as the optical window, as shown in Figure 1.

While the silicon window enables wafer-level assembly and robust packaging, its high refractive index (~3.4 in MWIR and LWIR) leads to strong Fresnel reflections intensity given by:

(1) $$R={\left(\frac{n_{si}-n_{air}}{n_{si}+n_{air}}\right)}^{2}$$

These effects substantially reduce transmission to the sensing element and limit system sensitivity. Therefore, high-performance anti-reflection coatings (ARCs) are critical to maximize IR throughput and ensure stable optical performance over a wide range of incidence angles.

### 2.2. Temperature-Dependent Electrical Transduction and Responsivity in CMOS–MOSFET TMOS Sensors

The TMOS detector operates as an active thermometric element whose electrical output is governed by subthreshold MOSFET conduction. In this regime, the drain current exhibits exponential sensitivity to temperature through mobility and threshold voltage variations, and can be expressed as follows [14]:

(2) $$I_{DS}(T)=I_{0}\;\exp\left(\frac{q\;\left(V_{GS}-V_{T}\left(T\right)\right)}{n\;k\;T}\right)$$

where n is the subthreshold ideality factor, $V_{GS}$ is the gate source voltage of the MOSFET transistor, and $V_{T}(T)$ is the threshold voltage. $V_{T}(T)$ for the NMOS decreases approximately linearly with temperature $\left(dV_{T}/dT<0\right)$ in CMOS–SOI processes. Absorbed infrared power raises the local device temperature by ΔT, shifting $V_{T}(T)$ and producing a corresponding increase in $I_{DS}$ with a temperature coefficient of current (TCC) on the order of several percent per Kelvin. The pixel responsivity in current-readout mode may be approximated as [14] follows:

(3) $$R_{i}\equiv \frac{\Delta I_{DS}}{P_{IR}}\propto \frac{\eta \;I_{DS}}{G_{th}}\left|\frac{q}{n\;k\;T}\left(\frac{dV_{T}}{dT}+\frac{V_{GS}-V_{T}}{T}\right)\right|$$

Any increase in optical efficiency η—for example, through a broadband anti-reflection metasurface—directly and linearly enhances electrical responsivity. Unlike passive absorbing films, the TMOS device provides internal gain through the exponential transfer from ΔT to drain current, allowing optical improvements at the silicon window to be transduced into measurable electrical amplification. This coupling between wafer-level optical transmission, thermal isolation, and MOSFET subthreshold sensitivity forms the basis for system-level performance enhancement in CMOS-integrated uncooled infrared sensors.

### 2.3. Concept of Moth-Eye Structures

**Figure 2:**
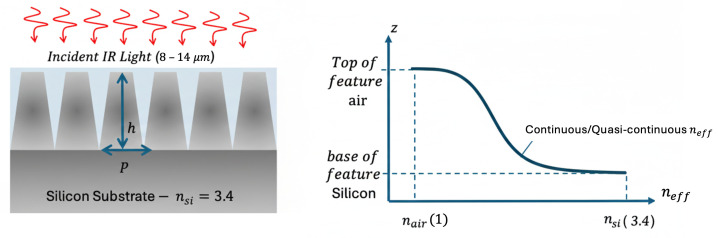
Schematic representation of the moth-eye inspired silicon surface and its effective refractive index profile. (**Left**) Conical features of height and pitch patterned on a silicon substrate are illuminated by incident infrared light in the 8–14 μm range. The graded geometry provides a gradual transition between air and silicon. (**Right**) Corresponding effective refractive index profile. The horizontal axis represents the effective refractive index ($n_{eff}$), and the vertical axis represents the spatial position (z) along the propagation direction. This illustrates the continuous index gradient from $n_{air}=1$ at the top to $n_{si}=3.4$ at the base, enabling optical broadband impedance matching.

Moth-eye surfaces consist of periodic arrays of subwavelength features typically cones, cylinders, or rectangular pillars, whose geometry defines the local silicon filling fraction. This fill-factor variation produces a continuous (or quasi-continuous) effective refractive index, smoothing the transition from $n_{air}$ to $n_{Si}$. For the infrared wavelengths of interest (8–14 μm), even relatively simple geometries such as cylindrical or rectangular pillars can approximate the graded-index behavior when the height-to-pitch ratio is sufficiently large. Figure 2 illustrates the moth-eye ARC concept. In Figure 2 (right), the vertical axis corresponds to the geometric depth (z) aligned with the structure shown in Figure 2 (left), while the horizontal axis plots the resulting variation in the effective refractive index ($n_{eff}$).

These structures inherently support broadband performance, wide angular tolerance, and polarization insensitivity, making them attractive for IR optical windows, sensors, and wafer-level packaging.

### 2.4. CMOS Manufacturing Compatibility

The implementation of moth-eye structures in wafer-level processed and packaged LWIR sensors requires consideration of both fabrication constraints and system-level performance metrics. This section discusses the selection of CMOS-compatible fabrication processes and compares the present work with prior studies in terms of manufacturing scalability, spectral range, and electrical sensor integration.

#### Fabrication Process Selection for CMOS-MEMS Integration

Wafer-level MEMS processing and packaging requires fabrication processes compatible with standard CMOS foundry workflows and high-vacuum hermetic environments. Table 1 compares the candidate nanofabrication techniques.

DRIE (deep reactive ion etching) emerges as the only process that meets all the requirements for production-scale CMOS–SOI–MEMS integration: it patterns silicon directly without deposited materials, maintains compatibility with vacuum packaging thermal budgets, and provides the anisotropic etching necessary for high-aspect-ratio subwavelength structures. This work specifically employs DRIE-compatible geometries (cylindrical pillars, aspect ratio ~0.5) that balance optical performance with manufacturing yield.

## 3. Analytical Models and Design Considerations

### 3.1. Effective Medium Approximation

In the deep subwavelength regime (λ≫P), the moth-eye pillar array may be modeled as an effective homogeneous layer characterized by a scalar refractive index under normal incidence. The effective index is primarily determined by the lateral silicon fill fraction.

A commonly used homogenization approach for two-phase composites is the Bruggeman effective medium approximation (EMA), which treats the constituent materials symmetrically and is often employed for pillar or porous geometries with moderate–high fill fractions. For a silicon–air composite, the Bruggeman relation is given by [17,18,19]:

(4) $$f\frac{n_{Si}^{2}-n_{eff}^{2}}{n_{Si}^{2}+2n_{eff}^{2}}+(1-f)\frac{n_{air}^{2}-n_{eff}^{2}}{n_{air}^{2}+2n_{eff}^{2}}=0$$

where $f=\pi r^{2}/P^{2}$ is the silicon fill fraction. For the geometry considered here, R=1 μm, P=3 μm, the fill fraction is f≈0.35, yielding a homogenized Bruggeman index $n_{EMT}\approx 1.55–1.6$. However, for finite-height pillars (h=1.5 μm), which are electrically thin in the LWIR band (h/λ∼0.1–0.2), the optical response is governed by the phase accumulation across the layer, and the lateral depolarization effects are minimal. In this case, the volume-averaged permittivity [20,21] is as follows:

(5) $$\varepsilon_{\mathrm{VA}}=f\varepsilon_{\mathrm{Si}}+(1-f)\varepsilon_{\mathrm{air}}$$

This provides a more accurate representation of the effective refractive index at normal incidence. This value closely matches the full-wave simulation results and captures the transmission magnitude and phase of the thin pillar layer more faithfully than the Bruggeman EMA.

### 3.2. Transfer Matrix Calculation

The moth-eye layer is discretized into a sequence of thin, homogeneous slab, each characterized by an effective refractive index derived from the homogenization model described in Section 3.1. The optical response of the resulting multilayer stack is computed using the standard transfer matrix formalism [22] (full details are provided in Appendix A), allowing efficient evaluation of reflection and transmission while explicitly accounting for Fabry–Perot interference arising from the finite thickness of the silicon substrate.

To obtain spectrally relevant performance metrics, the wavelength-dependent transmission is weighted by a 300 K blackbody emission spectrum and the detector responsivity across the LWIR band (8–14 μm). In addition, to enable a direct and realistic comparison with experimental measurements, the calculated spectra are power-averaged using a Lorentzian spectral response with δ=2THz [16,23]. This averaging accounts for the finite spectral resolution of the measurement system and suppresses high-frequency Fabry–Perot oscillations without altering the underlying broadband transmission trend.

This semi-analytical transfer matrix method (TMM) framework enables the rapid exploration of the design parameter space and provides physical insights, serving as an efficient precursor to full-wave FDTD simulations.

### 3.3. Diffraction Analysis and Period Selection

While the effective medium theory captures optical impedance matching in the subwavelength regime, it does not account for diffractive scattering that occurs when λ becomes comparable to or smaller than the period P. For a periodic array, the grating equation predicts the emergence of diffraction orders [24,25]:

(6) $$sin\theta_{m}=\frac{m\lambda }{P}-sin\theta_{i}$$

where m is the diffraction order and $\theta_{i}$ is the incidence angle. At normal incidence ($\theta_{i}\;=\;0$), first-order diffraction (m = ±1) propagates when λ < P, redirecting energy away from the forward direction and reducing on-axis transmission.

For our design targeting the LWIR band (8–14 μm), the period P = 3 μm was chosen to satisfy λ/P > 2.7 across most of the spectrum, ensuring effective medium behavior with minimal diffractive losses. At the short-wavelength edge (λ ≈ 6–7 μm), first-order diffraction begins to emerge but remains weak due to the relatively low pillar height (h = 1.5 μm in our design). This design trade-off balances fabrication feasibility—smaller periods require finer lithography and higher aspect ratios—with optical performance.

The transition from diffractive to effective medium regimes is rigorously evaluated using full-wave FDTD simulations with farfield projections (Section 4.4), which capture the complete angular scattering behavior beyond the capabilities of scalar diffraction theory.

## 4. Numerical Simulations

**Figure 3:**
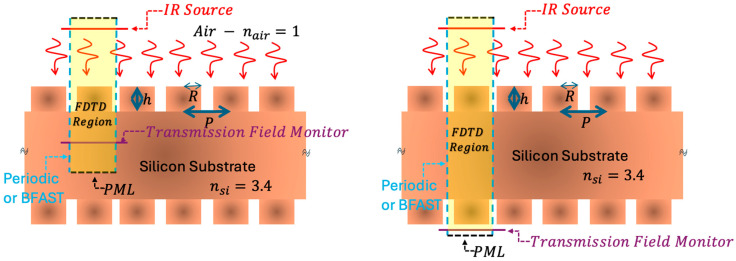
FDTD simulation configurations. (**Left**) Single-surface geometry: cylindrical silicon pillars (radius *R*, height *h*, period *P*) at the air–silicon interface. (**Right**) Full wafer window: finite-thickness silicon slab with pillars on one or both sides. Periodic boundary conditions applied laterally or Broadband Fixed Angle Source Technique (BFAST) for the non-normal incident of source light; perfectly matched layers (PMLs) terminate vertically. Transmission monitors (dashed boxes) measure transmitted power.

Full-wave electromagnetic simulations were performed using the finite-difference time-domain (FDTD) method (Lumerical FDTD Solutions [26]) to evaluate moth-eye optical performance beyond effective medium approximations. Figure 3 illustrates the two simulation geometries employed: (left) single-surface pillar arrays for parametric optimization, and (right) finite-thickness silicon slabs with single- or double-sided patterning representing the actual device geometry. The single-surface configuration (air → moth-eye surface → semi-infinite silicon) enables isolation of the graded-index behavior at a single interface without Fabry–Perot effects from the finite substrate. This allows us to quantify the intrinsic impedance-matching contribution of the moth-eye pillars and provides a computationally efficient precursor to the full wafer geometry. The latter (finite silicon slab with patterned surfaces) is then used to evaluate complete system transmission for single- and double-sided patterning.

### 4.1. Simulation Setup

The computational domain corresponded to a single unit cell with periodic boundary conditions in the lateral directions and perfectly matched layers (PMLs) along the propagation axis. Silicon was modeled using measured refractive index data using Palik data (6–14 μm, $n_{Si}\;\approx \;3.4$ in LWIR) [27], with air as *n* = 1. Mesh refinement captured subwavelength features near the pillars (*R* = 1 μm, *P* = 3 μm). A broadband plane wave source (6–14 μm) illuminated from air was assumed. Simulations included both polarizations and various incident angles, with frequency-domain monitors recording transmission and far-field projection monitors computing angular scattering.

### 4.2. Parametric Analysis

**Figure 4:**
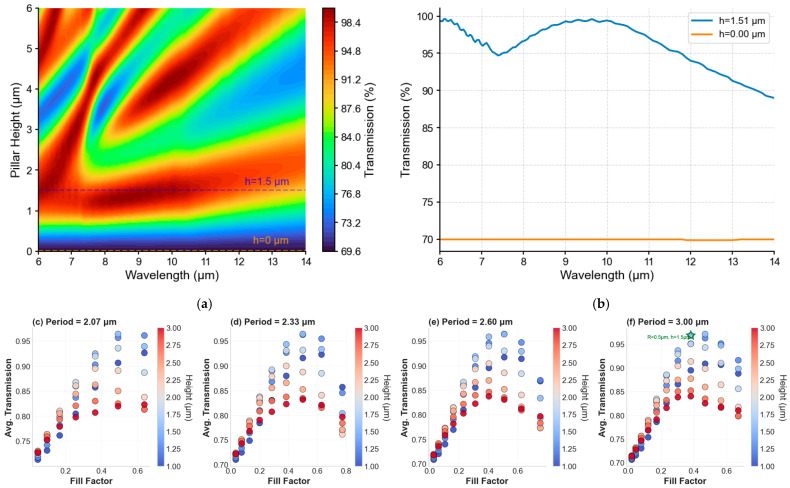
Pillar height parametric sweep for single side air–silicon interface. (**a**) Transmission contour map vs. height and wavelength (*R* = 1 μm, *P* = 3 μm). (**b**) Spectra for *h* = 0 (bare) and *h* = 1.5 μm (optimized), showing significant LWIR enhancement. (**c**–**f**) Fill factor versus wavelength-weighted average transmission in the 8–14 μm band for four pillar periods: (**c**) P ≈ 2.07 μm, (**d**) P ≈ 2.33 μm, (**e**) P ≈ 2.67 μm, (**f**) P = 3.00 μm. Each marker corresponds to a simulated geometry; the vertical axis shows the transmission averaged over 8–14 μm, the horizontal axis is the structure fill factor (pillar area over the period area, $\pi R^{2}/P^{2}$, and marker color encodes pillar height (colormap: coolwarm). In panel (**f**) a star marks the simulation closest to the target device (radius ≈ 1.0 μm, height ≈ 1.5 μm). Panels (**c**–**f**) illustrate how transmission depends on fill factor and height across periods, showing that fill-factor/radius changes are the dominant tuning parameter while height mainly introduces secondary spectral features. In this work, the green star in Panel (**f**) indicates the parameter values used for the fabricated pillars.

Figure 4 presents the parametric analysis for the varying periods, heights, and radii of the pillar features. Figure 4a shows transmission versus pillar height and wavelength for *R* = 1 μm, *P* = 3 μm under normal incidence. Bare silicon (*h* = 0) exhibits ~54% transmission due to Fresnel reflection. Transmission improves with increasing height, peaking near *h* ≈ 1.5 μm across 8–14 μm due to the gradual effective index profile. Figure 4b compares *h* = 0 and *h* = 1.5 μm, showing ~30% relative enhancement at 10 μm. This geometry was selected for fabrication, balancing optical performance with process feasibility.

Figure 4c–f show how average transmission efficiency (8–14 μm) varies with pillar fill factor for four different periods (2.07–3.00 μm). The similar transmission behavior across all periods when plotted against fill factor validates the effective medium approximation, demonstrating that optical performance is governed primarily by the silicon filling fraction rather than absolute feature size. Consequently, the key design parameters are the minimum period and gap achievable by the fabrication facility, with fill factor and pillar height then optimized to achieve the target effective refractive index profile. Cylindrical pillars were selected over rectangular or sharp-edged geometries because they are more reliably fabricated and less prone to edge damage or collapse during DRIE processing.

### 4.3. Angular and Polarization Performance

**Figure 5:**
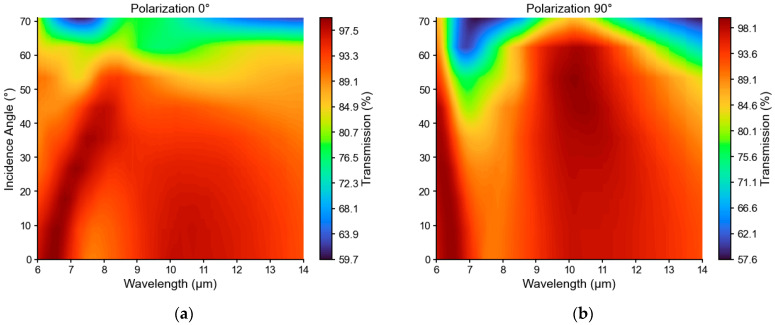
Angular and polarization performance for single side air–silicon interface. Transmission vs. wavelength and incident angle for (**a**) 0° and (**b**) 90° polarization. High transmission maintained across LWIR band for angles up to ~50° with negligible polarization sensitivity.

Moth-eye structures exhibit wide angular acceptance and weak polarization dependence compared to thin-film coatings. Figure 5 shows transmission versus incident angle (0–70°) and wavelength for both polarizations (*R* = 1 μm, *P* = 3 μm, *h* = 1.5 μm). Transmission exceeds 80% across the LWIR band for angles up to ~50°, with minimal polarization dependence (<3% deviation below 60°). This behavior arises from cylindrical symmetry and subwavelength operation, confirming robust performance for unpolarized thermal radiation across typical sensor fields of view (≤30° half-angle).

### 4.4. Far-Field Diffraction Analysis

**Figure 6:**
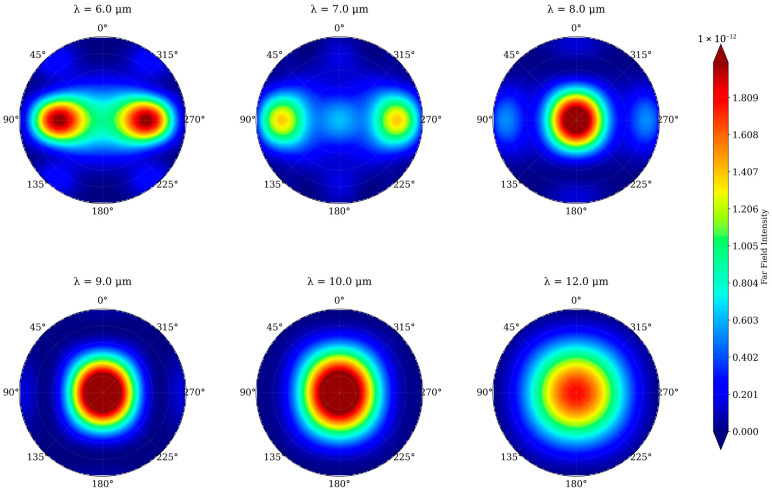
Far-field transmission patterns for single side air–silicon interface at six wavelengths (6–12 μm) for *R* = 1 μm, *P* = 3 μm, *h* = 1.5 μm. Radial axis: polar angle (0–90°); color scale: normalized intensity. Transition from diffractive (*λ* < 8 μm) to effective medium regime (*λ* > 9 μm) confirms graded-index AR behavior in the LWIR band.

The wavelength-to-period ratio (*λ*/*P*) governs the transition from diffractive to effective medium behavior. Figure 6 shows far-field patterns for *λ* = 6–12 μm (*R* = 1 μm, *P* = 3 μm, *h* = 1.5 μm). At *λ* = 6–7 μm (*λ*/*P* ≈ 2–2.3), strong first-order diffraction lobes appear at ±90°. At *λ* = 9–12 μm (*λ*/*P* > 3), near-circular symmetry indicates effective medium behavior with minimal angular scattering. The transition occurs at *λ* ≈ 8–9 μm, aligned with the LWIR band edge, validating the 3 μm period choice. Residual diffraction below 8 μm falls outside the target detection range.

### 4.5. Full System Simulation: Single- vs. Double-Sided Patterning

**Figure 7:**
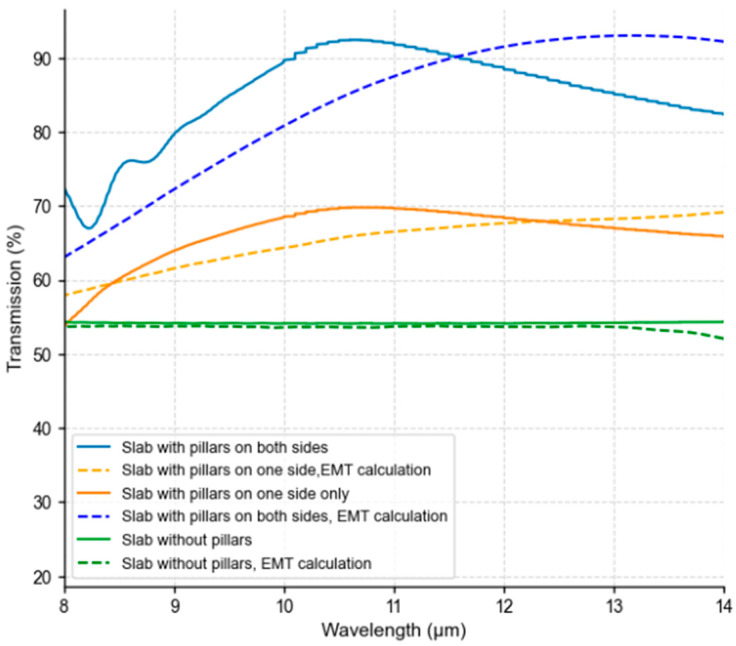
Full wafer window transmission: FDTD and effective medium theory (EMT) comparison. Transmission spectra for 500 μm silicon slab with various configurations: bare (green), single-sided pillars (orange), and double-sided pillars (blue). Solid lines: FDTD simulations; dashed lines: EMT predictions. Double-sided patterning achieves 85.11% band-averaged transmission (8–14 μm), a 57.06% improvement over bare silicon. Close agreement between FDTD and EMT validates effective medium modeling in the LWIR regime.

The complete wafer window structure (50 μm thick silicon slab) was simulated in three configurations: bare, single-sided pillars, and double-sided pillars. The results are presented in Figure 7. Single-sided patterning increased band-averaged transmission (8–14 μm) from 54.19% to 66.47% (+12.28, 22.65% relative). Double-sided patterning achieved 85.11% (+30.92, 57.06% relative). These gains directly enhance sensor responsivity (Section 7.3).

An important practical consideration for double-sided patterning is whether the relative alignment between top and bottom pillar arrays affects performance. In the subwavelength regime, alignment is not critical. When the period is much smaller than the wavelength, the electromagnetic field cannot resolve individual pillars and instead experiences a homogenized refractive index determined by the fill fraction (Section 3.1). The optical response is governed by the vertical index gradient rather than lateral periodicity—as evidenced by the close agreement between FDTD and EMT in Figure 7, which both treat the layers as homogeneous media.

FDTD simulations comparing aligned versus arbitrarily rotated pillar configurations confirmed transmission differences < 0.5% across 8–14 µm. This alignment insensitivity is a major manufacturing advantage: both surfaces can be patterned independently without precision alignment tools, significantly reducing fabrication complexity and cost compared to approaches requiring layer-to-layer registration (e.g., multilayer thin films or photonic crystals).

## 5. Fabrication

**Figure 8:**
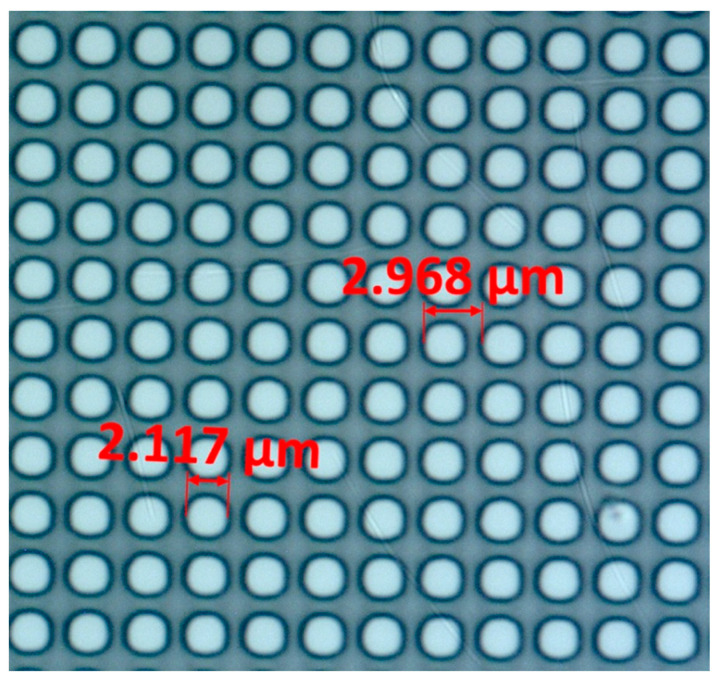
Optical micrograph showing the periodic cylindrical pillar array with a measured diameter of approximately 2.12 μm and a pitch of 2.97 μm.

The anti-reflection structures were fabricated on high-purity float-zone (FZ) silicon wafers (diameter: 50.8 mm, thickness: 381 ± 20 μm, ⟨100⟩ orientation, resistivity: 2.25–3.75 Ω · cm, n-type phosphorus doping). The moth-eye pattern consisted of cylindrical pillars with a diameter of approximately 2.12 μm and a pitch of 2.97 μm, as verified by optical microscopy (Figure 8).

Patterns were defined using direct laser writing, followed by resist development to form the etch mask. The structures were transferred into silicon using inductively coupled plasma deep reactive ion etching (ICP-DRIE), achieving an etch depth of about 1.5 μm with vertical sidewalls and uniformity across the wafer.

To enable FTIR transmission measurements, the patterned wafers were diced into 10 × 10 mm samples using a precision dicing system. Cylindrical pillars were selected over conical geometries due to their superior manufacturability and robustness under standard plasma etching conditions. Challenges included maintaining uniformity and preventing pillar collapse during etching. Future work will focus on double-sided patterning.

## 6. Optical Characterization (FTIR)

**Figure 9:**
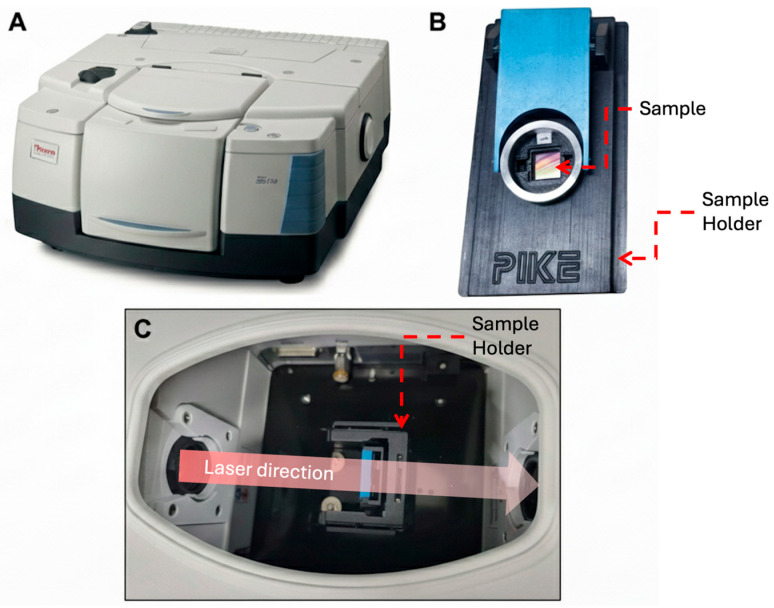
FTIR transmission measurement setup. (**A**) Thermo Scientific Nicolet iS50 FTIR spectrometer configured for transmission measurements. (**B**) transmission samples holder with the sample inserted in the central aperture of the holder. (**C**) Internal view of the spectrometer showing the sample holder positioned along the optical path and the direction of the incident infrared beam.

Transmission measurements were carried out using a Thermo Fisher Nicolet iS50 FTIR spectrometer (Thermo Fisher Scientific, Waltham, MA, USA) equipped with a dedicated transmission module [28]. The measurements covered the 6–14 μm spectral range at normal incidence, with a spectral resolution of 5 nm. Samples were mounted in the transmission accessory to ensure consistent alignment as presented in Figure 9. Background correction was performed using an air reference, and all spectra were normalized to a polished, unpatterned silicon wafer to isolate the effect of the nanostructured anti-reflection coating.

**Figure 10:**
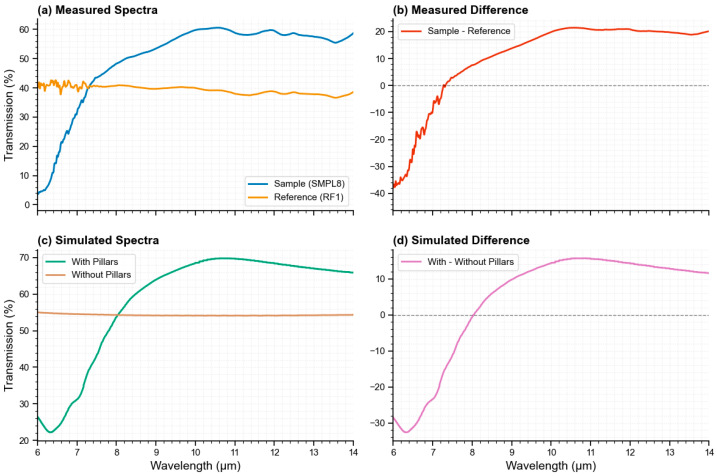
Transmission spectra and differential analysis of silicon samples single side pillars. (**a**) Spectral transmission measurements for the patterned sample and the un-patterned reference wafer, plotted as percent transmission versus wavelength (μm); (**b**) pairwise difference spectrum (sample spectra minus reference spectra), highlighting regions where the patterned sample exhibits higher transmission or lower transmission than the reference; (**c**) spectral transmission simulation results for the patterned sample and the un-patterned reference wafer, plotted as percent transmission versus wavelength (μm); (**d**) pairwise difference spectrum (simulated patterned minus bare wafer).

Figure 10 presents transmission spectra and differential analysis across the 6–14 μm range, comparing bare silicon and moth-eye patterned samples. Figure 10a,c show absolute transmission for experimental and simulated configurations, respectively, while Figure 10b,d display the corresponding differential spectra (patterned minus bare), directly visualizing the wavelength-dependent enhancement provided by the nanostructures. The spectral profiles reveal several key features: both experimental and simulated data exhibit peak transmission in the 9–12 μm region, with maximum enhancement occurring near 10–11 μm as shown prominently in the differential plots. The enhancement bandwidth (defined as the region where differential transmission exceeds +10 percentage) spans approximately 8–13 μm, covering the majority of the LWIR atmospheric window. Notably, transmission decreases sharply below ~8 μm in patterned samples, with the differential spectra approaching zero or even becoming slightly negative in this regime, indicating that the anti-reflection benefit diminishes when diffractive scattering becomes dominant.

The reduced transmission observed below ~8 μm is attributed to first-order diffraction, which occurs when the wavelength becomes comparable to the pillar period (λ ≲ P). Consistent with the far-field analysis in Section 4.4 (Figure 6), the structure transitions into an effective medium regime when λ/P ≳ 2.5–3, corresponding to ~7.5–9 μm for P ≈ 3 μm. Above this threshold, diffractive losses are suppressed and broadband transmission enhancement is achieved across the LWIR band, as evidenced by the consistently positive differential spectra in Figure 10b,d throughout the 8–14 μm range. The close spectral correspondence between experimental (Figure 10b) and simulated (Figure 10d) differential curves—both showing similar peak wavelengths, spectral widths, and magnitude profiles—validates the electromagnetic modeling approach and confirms that the fabricated structures exhibit the designed gradient-index anti-reflection behavior.

## 7. LWIR Transmission Analysis (8–14 μm)

The optical performance of moth-eye anti-reflection coatings was evaluated across the long-wave infrared (LWIR) band using numerical simulations, analytical modeling, and experimental measurements. These results were correlated with the expected responsivity enhancement in CMOS–SOI–MEMS thermal IR sensors.

### 7.1. Numerical Predictions: FDTD and EMT Analysis

Full-wave finite-difference time-domain (FDTD) simulations quantified the transmission improvement provided by subwavelength pillar arrays on silicon windows. The baseline bare silicon wafer exhibited an average LWIR transmission of 54.19%. Introducing a single-sided moth-eye structure increased transmission to 66.47%, while a double-sided configuration achieved 85.11%. These correspond to absolute gains of +12.28% (22.65% relative) for single-sided and +30.92% (57.06% relative) for double-sided designs. The large improvement in the double-sided case underscores the importance of optical impedance matching at both air–silicon interfaces for maximizing throughput.

Analytical predictions using effective medium theory (EMT) provided rapid design-space estimates, yielding normalized transmission factors of approximately 65% for single-sided and 84% for double-sided configurations. While EMT does not capture diffractive effects, its agreement with FDTD trends validates its utility for preliminary optimization.

### 7.2. Experimental Validation: FTIR Measurements

Fabricated samples were characterized using Fourier-transform infrared (FTIR) spectroscopy. The reference bare silicon sample exhibited an average transmission of 38.84%, whereas the patterned sample achieved 56.96%, corresponding to an improvement of +18.12 (46.67% relative). This experimental gain confirms the effectiveness of the moth-eye design under practical fabrication constraints and demonstrates compatibility with wafer-scale MEMS processes. Minor discrepancies between simulation and measurement are attributed to fabrication tolerances, surface roughness, and finite pillar height.

### 7.3. System-Level Impact on Sensor Responsivity

The responsivity of TMOS-based thermal IR sensors scales linearly with the optical efficiency of the window (see Equation (3)). Consequently, the measured transmission improvement translates directly into a responsivity boost of ~46.67% for the fabricated sample compared to a standard silicon cap. Simulation results predict boosts of 22.65% for single-sided and 57.06% for double-sided configurations. This coupling between optical and electrical performance establishes moth-eye coatings as active enablers of next-generation uncooled infrared sensors, rather than passive optical accessories.

The results are summarized in Table 2.

### 7.4. Comparison of Moth-Eye Anti-Reflection Coating Studies for Infrared Applications

To contextualize the contributions of this work, Table 3 compares our results with recent developments in infrared moth-eye structures. While prior works have explored moth-eye windows for thermal imaging, our work is distinguished by the presentation of an integrated end-to-end framework that bridges the gap between optical metasurface modeling and active sensor responsivity.

The architectural choice is critical for performance reliability in industrial applications. Papatzacos et al. [7] demonstrated an innovative and sophisticated “ball-on-pole” design utilizing the GOPHER process flow. While their simulations predicted a ~10% increase in transmission, the experimental improvement was limited to approximately 5%. As the authors noted, this discrepancy is likely due to the high sensitivity of complex, non-vertical geometries to surface roughness and scattering losses which are challenging to account for in idealized simulations. This suggests that while such advanced geometries offer significant theoretical potential, they may require further maturation in manufacturing precision and high-fidelity metrology to achieve their full performance in a production environment.

In contrast, the straight-pillar architecture employed in this work—consistent with established publications (e.g., Brewer et al. [29])—is currently preferred for wafer-scale and WLP-MEMS sensors. By utilizing a more robust and vertical geometric profile achievable through standard deep reactive ion etching (DRIE).

## 8. Discussion

This work presents a comprehensive investigation of moth-eye anti-reflection coatings for silicon wafer-level packaging in LWIR thermal sensors, bridging optical theory, numerical design, microfabrication, and system-level performance analysis. The results demonstrate that subwavelength pillar arrays can achieve substantial transmission enhancement across the 8–14 μm atmospheric window, with measured improvements of approximately 18.1% (46.67% relative increase) over bare silicon for single-sided patterning. FDTD simulations predict that double-sided patterning can push this enhancement to 30.9% (57.06% relative increase). These gains translate directly to proportional increases in sensor responsivity, offering a pathway to improved signal-to-noise ratio.

Several key findings emerge from this study. First, the transition from diffractive to effective medium behavior occurs predictably when the wavelength-to-period ratio exceeds approximately 2.5–3, validating the design criterion that informed our 3 μm period selection. Farfield scattering analysis confirms that higher-order diffraction is effectively suppressed across most of the LWIR band, with residual diffraction at shorter wavelengths (6–7 μm) falling outside the target spectral window.

The measured absolute transmission values are systematically lower than FDTD predictions by approximately 10–14%. This offset likely arises from several non-idealized factors: (1) surface roughness and micron-scale imperfections, (2) absorption from trace impurities in float-zone silicon, and (3) scattering from fabrication non-uniformities. Importantly, the relative enhancement due to pillar patterning is consistent between simulation (+12–16%) and experiment (+18.1%), validating the design principle. The lower bare silicon transmission (~40% vs. ~54% predicted) suggests the reference sample experienced degradation or contamination, making the measured enhancement even more significant.

**Figure 11:**
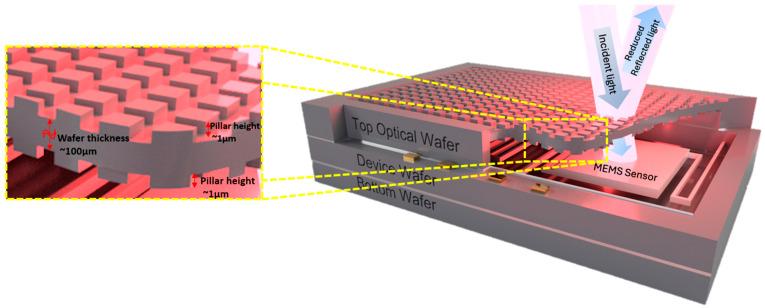
Illustrates the proposed wafer-level package architecture showing double-sided moth-eye patterning on both surfaces of the top optical window, enabling enhanced incident light transmission to the underlying MEMS sensor while maintaining hermetic vacuum sealing through wafer bonding.

Second, the agreement between FDTD predictions and FTIR measurements—both showing transmission peaks near 10–11 μm and band-averaged enhancements of 22.65% (FDTD, single-sided) versus 46.67% (experimental)—validates the modeling framework. The higher experimental improvement likely reflects differences between the idealized bare silicon baseline used in simulations and the actual reference sample measured in FTIR, which may have exhibited surface oxidation or contamination that reduced its transmission relative to pristine silicon. Third, the demonstration of double-sided patterning in simulation (85.11% average transmission) establishes the theoretical potential of symmetric AR treatment on thin silicon windows, a critical requirement for maximizing optical throughput in vacuum-sealed sensor packages. Applying moth-eye structures to both surfaces of the silicon optical window, as illustrated in Figure 11, significantly enhances sensor performance.

The close correspondence between EMT predictions and full-wave FDTD results (Figure 7) confirms that effective medium theory provides a reliable first-order design tool for rapid parameter exploration, particularly in the longer-wavelength regime where λ/P ratios are largest. However, EMT slightly overestimates transmission at shorter wavelengths where diffractive effects become more pronounced, underscoring the value of full-wave validation for final design optimization.

The measured transmission, while significantly improved, remains below the theoretical maximum for an ideal gradient-index profile, primarily due to the discrete (single-layer) nature of the pillar array rather than a continuous taper [6,8]. Additionally, the current design targets normal incidence; while moth-eye structures inherently exhibit wide angular tolerance, quantitative characterization of off-axis performance was not performed and remains a subject for future investigation.

The experimental validation of the modeling framework—spanning from effective medium approximations through full-wave FDTD to FTIR measurements—confirms the viability of this approach for wafer-level manufacturing. As outlined in the Introduction, conventional thin-film ARCs face significant integration challenges in MEMS packaging, including thermal budget limitations and material interface compatibility. The demonstrated compatibility of moth-eye structures with standard silicon processing, combined with the measured 46.67% responsivity enhancement, validates the substrate patterning approach for practical deployment. The simulated 57.06% improvement for double-sided configurations represents a clear pathway to further performance gains. Unlike narrow-band interference coatings, the broadband nature of the moth-eye response—maintained across the full 8–14 μm window with minimal angular sensitivity—directly addresses the requirements of uncooled thermal imaging systems. While fabrication complexity (deep etching, high-aspect-ratio lithography) currently exceeds that of vacuum deposition, recent advances in nanoimprint lithography and scalable plasma etching [30,31,32] suggest this gap is narrowing, particularly for high-volume sensor production where the substrate-integrated approach eliminates post-processing material deposition steps.

## 9. Conclusions and Future Work

This work establishes a comprehensive framework for the design, fabrication, and characterization of moth-eye anti-reflection coatings on silicon wafer-level packages for LWIR thermal CMOS–SOI–MEMS sensors. Through the integration of effective medium theory, full-wave FDTD simulations, and experimental FTIR validation, we demonstrate that subwavelength pillar arrays can enhance transmission by 18.1% across the 8–14 μm atmospheric window, translating to a 46.67% responsivity boost in TMOS-based thermal detectors. The close agreement between analytical predictions, numerical simulations, and measured spectra validates the modeling approach and confirms the feasibility of wafer-scale processing and packaging implementation. Critically, this study prioritizes design guidelines and fundamental optical analysis rather than ultimate performance optimization—the 3 μm period employed here serves as a proof-of-concept demonstration that balances fabrication accessibility with sufficient subwavelength behavior in the target LWIR band, corresponding to manufacturing reality.

Future work will focus on several key directions to advance this technology toward practical deployment:

Double-sided patterning: FDTD simulations predict that symmetric moth-eye structuring on both surfaces of the optical window can increase transmission to 85.11% (30.9% above bare silicon), corresponding to a 57.06% responsivity enhancement. Implementing double-sided pillars on production wafer-level packages represents a high-impact pathway to significantly boost sensor performance with minimal changes to existing MEMS architectures.

Reduced period for broadband optimization: The 3 μm period used in this proof-of-concept study was selected to ensure robust fabrication and clear demonstration of effective medium behavior. Future iterations will explore smaller periods (1.5–2 μm) to push deeper into the subwavelength regime across the entire LWIR band, suppressing residual diffraction at shorter wavelengths and achieving more uniform transmission from 8–14 μm.

Advanced structural characterization: Scanning electron microscopy (SEM) and atomic force microscopy (AFM) will be employed to quantify pillar geometry, sidewall profiles, and surface roughness. Cross-sectional analysis will enable correlation between fabrication parameters and optical performance, guiding process optimization for high-aspect-ratio structures.

System-level sensor prototyping and testing: While this work quantifies the optical transmission enhancement and its predicted impact on responsivity, direct measurement of device-level electrical output remains essential. Future efforts will integrate moth-eye windows into fully packaged TMOS sensors and characterize actual responsivity improvements, noise-equivalent temperature difference (NETD), and thermal time constants under operational conditions.

Collectively, these advances will transition moth-eye anti-reflection microstructures from a laboratory demonstration to a production-ready technology for next-generation thermal infrared imaging systems.

## Figures and Tables

**Table 1:** Comparison of fabrication processes for wafer-level moth-eye anti-reflection structures.

Process	CMOS Foundry Availability	Achievable Shapes	Aspect Ratio Control	Wafer Scale Uniformity	Vacuum Package Compatible	Status for WLP-MEMS
DRIE/RIE	Standard	Cylindrical, rectangular, hexagonal pillars	High (>5:1)	Excellent	Native Silicon	Production-ready
Nanoimprint Lithography (NIL)	Research only	Any shape (mold-limited)	Limited by demolding	Variable	Polymer outgassing	Laboratory demonstration
Laser Ablation	Non-standard	Conical, gaussian profiles	Moderate	Serial process	Surface damage/recast	Mainly for Prototype
Laser Interference	Specialized	Sinusoidal, conical	Limited	Alignment challenges	With post-etching	Research tool
Grayscale Lithography + RIE	Specialized resists	Conical, arbitrary 3D	Good	Good	Native silicon	Emerging technology

**Table 2:** Results summary.

Configuration	Transmission (8–14 μm)	Improvement vs. Bare	Responsivity Boost
Bare Silicon (Simulation)	54.19%	—	—
Single-Sided Pillars (FDTD Simulation)	66.47%	+12.28%	22.65%
Double-Sided Pillars (FDTD Simulation)	85.11%	+30.92%	57.06%
Reference Sample (FTIR Measurement)	38.84%	—	—
Fabricated Pillars (FTIR Measurement)	56.96%	+18.12%	46.67%

**Table 3:** Comparison of Infrared Moth-Eye Coating Performance and Contributions to Sensor Systems.

Feature	Papatzacos et al. [7]	Brewer et al. [29]	This Work
Pattern Pitch	3.7 μm	2.0 μm	3.0 μm
Spectral Target	12–16 μm	7–14 μm	8–14 μm
Moth-eye shape	“Ball-On-Pole” pillars	Cylindrical pillars	Cylindrical pillars
Key Result	5% Transmission Uplift	40% Transmission Uplift	46.7% Responsivity Boost
Contribution	Novel shape with isotropic and anisotropic etching.	Double sided pillar fabrication and Demonstrated image-preserving	End-to-end design consideration for WLP IR sensor link: Modeling, simulation, validation and impact on sensor responsivity

**Figure A1:**
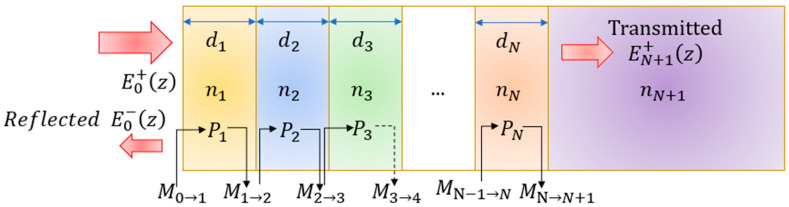
Transfer matrix method for homogenized layers illustration.

## Data Availability

Data is available upon reasonable request from the corresponding author.

## Abbreviations

The following abbreviations are used in this manuscript:

ARC	anti-reflection coating
CMOS	complementary metal oxide semiconductor
EMA	effective medium approximation
EMT	effective medium theory
FDTD	finite-difference time-domain
FTIR	Fourier-transform infrared
ICP-DRIE	inductively coupled plasma–deep reactive ion etching
IR	infrared
LWIR	long-wave infrared
MEMS	micro-electro-mechanical systems
MOSFET	metal oxide semiconductor field-effect transistor
MWIR	mid-wave infrared
NETD	noise-equivalent temperature difference
PML	perfectly matched layer
SEM	scanning electron microscopy
SOI	silicon-on-insulator
TCC	temperature coefficient of current
TE	transverse electric
TM	transverse magnetic
TMM	transfer matrix method
TMOS	transistor-based metal oxide semiconductor (thermal sensor)
WLP	wafer-level package

## Appendix A. Transfer Matrix Method for Homogenized Layers

Following layer homogenization, the optical response of the moth-eye structures can be efficiently analyzed using the Transfer Matrix Method (TMM). This method calculates reflection, transmission, and absorption for multilayered structures by tracking electric field amplitudes, especially useful when layers are characterized by effective parameters like refractive index ($n_{eff}$).

TMM’s core involves relating forward-propagating ($E^{+}$) and backward-propagating ($E^{-}$) electric field amplitudes at each layer boundary. For a plane wave in the z-direction, these amplitudes are represented as a column vector:

(A1) $$\left(\begin{array}{c} E_{j}^{+}\left(z\right)\\ E_{j}^{-}\left(z\right) \end{array}\right)$$

where $E_{j}^{+}\left(z\right)$ and $E_{j}^{-}\left(z\right)$ are the forward- and backward-propagating electric field amplitudes in layer j at z. Propagation and interface matrices govern their relationships.

Interface Matrix: The interface matrix $M_{j\to j+1}$ transforms electric field amplitudes across the boundary from layer j to j + 1. It relates amplitudes in layer j + 1 to those in layer j, based on Fresnel reflection ($r_{j\to j+1}$) and transmission ($t_{j\to j+1}$) coefficients:

(A2) $$M_{i\to i+1}=\frac{1}{1-r_{i\to i+1}}\left(\begin{array}{cc} 1 & -r_{i\to i+1}\\ -r_{i\to i+1} & 1 \end{array}\right)$$

Identities include $r_{b\to a}=-r_{a\to b}$ and $t_{a\to b}t_{b\to a}=1-r_{a\to b}^{2}$. For normal incidence, coefficients use impedances: $r_{j\to j+1}=(\eta_{j+1}-\eta_{j})/(\eta_{j+1}+\eta_{j})$ and $t_{j\to j+1}=2\eta_{j+1}/\eta_{j+1}+\eta_{j}$. Oblique incidence uses appropriate TE or TM impedances. Where $\eta_{j}$ is the optical impedance of layer j

Propagation Matrix: The propagation matrix $P_{j}$ describes electric field amplitude changes through a homogeneous layer j of thickness $d_{j}$, relating amplitudes at its front to its back interface. For the phase thickness, $\delta_{j}$:

(A3) $$P_{j}=\left(\begin{array}{cc} e^{-\frac{j\delta }{2}} & 0\\ 0 & e^{\frac{j\delta }{2}} \end{array}\right)$$

where $\delta_{j}=\frac{2\pi }{\lambda }n_{j}d_{j}cos\theta_{j}$ is the layer’s phase thickness, λ is free-space wavelength, $n_{j}$ is layer j’s effective refractive index, and $\theta_{j}$ is its angle of refraction.

Total Transfer Matrix: For an N-layer stack (incident medium 0, layers 1 … N, substrate N + 1), the total transfer matrix Mtotal is the product of alternating interface and propagation matrices:

(A4) $$\begin{array}{r} M_{total}=M_{N\to N+1}P_{N}M_{N-1\to N}P_{N-1}\ldots M_{1\to 2}P_{1}M_{0\to 1}\\ =M_{N\to N+1}\left(\prod_{j=1}^{N}P_{j}M_{j-1\to j}\right)=\left(\begin{array}{cc} A & B\\ C & D \end{array}\right) \end{array}$$

This matrix relates electric field amplitudes at the input plane to those at the output plane:

(A5) $$\left(\begin{array}{c} E_{N+1}^{+}\left(z\right)\\ E_{N+1}^{-}\left(z\right) \end{array}\right)=\left(\begin{array}{cc} A & B\\ C & D \end{array}\right)\left(\begin{array}{c} E_{0}^{+}\left(z\right)\\ E_{0}^{-}\left(z\right) \end{array}\right)$$

Here, $E_{0}^{+}\left(z\right)$ and $E_{0}^{-}\left(z\right)$ are incident and reflected amplitudes in the incident medium, while $E_{N+1}^{+}\left(z\right)$ and $E_{N+1}^{-}\left(z\right)$ are in the substrate. The transfer matrix method is illustrated in Figure A1.

Total reflection and transmission: For a structure with a semi-infinite substrate, $E_{N+1}^{-}\left(z\right)$ = 0. Using this with $M_{total}:$

From 0 = C$E_{0}^{+}$ + D$E_{0}^{-}$ the reflected field is $E_{0}^{-}\;$= $-\frac{C}{D}E_{0}^{+}$. The total reflection coefficient for the electric field, $r_{total},$ is:

(A6) $$r_{total}=\frac{E_{0}^{-}}{E_{0}^{+}}=-\frac{C}{D}$$

The intensity reflection is equal to $R=|r_{total}{|}^{2}$, and the intensity transmission is given by:

(A7) $$T_{total}=\frac{R\left(n_{N+1}cos\theta_{N+1}\right)}{R\left(n_{0}cos\theta_{0}\right)}{\left|t_{total}\right|}^{2}$$
